# Monitoring and Evaluating Progress towards Universal Health Coverage in Brazil

**DOI:** 10.1371/journal.pmed.1001692

**Published:** 2014-09-22

**Authors:** Mauricio L. Barreto, Davide Rasella, Daiane B. Machado, Rosana Aquino, Diana Lima, Leila P. Garcia, Alexandra C. Boing, Jackson Santos, Juan Escalante, Estela M. L. Aquino, Claudia Travassos

**Affiliations:** 1 Instituto de Saúde Coletiva, Federal University of Bahia, Salvador, Brazil; 2 National Institute in Science, Technology and Innovation in Health (CITECS), Salvador, Brazil; 3 Instituto de Pesquisa Econômica Aplicada, Brasília, Brazil; 4 Federal University of Santa Catarina, Floranopólis, Brazil; 5 Secretaria de Vigilância a Saúde, Ministry of Health, Brasília, Brazil; 6 Instituto de Comunicação e Informação Científica e Tecnológica em Saúde (ICICT)/FIOCRUZ, Rio de Janeiro, Brazil

## Abstract

This paper is a country case study for the Universal Health Coverage Collection, organized by WHO. Mauricio Barreto and colleagues illustrates progress towards UHC and its monitoring and evaluation in Brazil.

*Please see later in the article for the Editors' Summary*

This paper is part of the PLOS Universal Health Coverage Collection. This is the summary of the Brazil country case study. The full paper is available as Supporting Information file Text S1.

## Background

Brazil is a large and heterogeneous country, which has undergone rapid economic and social improvements, including changes in major social determinants of health and in the organization of the health system [1],[2]. Universal health coverage (UHC) is a fundamental principle of the Brazilian Unified Health System (SUS) targeted to implement the constitutional right (established by the Constitution of 1988) to health for all Brazilian citizens.

## Universal Health Coverage: The Policy Context

Since 1988, Brazil has been making efforts to develop the SUS, aiming at providing comprehensive and universal care, at the preventive and curative level, through decentralized management and provision of health services. The SUS provides care at the primary, secondary, and tertiary levels, and promotes community participation. However, the Brazilian health system includes an intricate public-private mix, and approximately one-quarter of the Brazilian population (the wealthiest sector) is covered by private health plans [1].

## Monitoring and Evaluation for UHC

The SUS has made advances in decentralized management processes, involving intermanagerial committees and negotiation mechanisms between federal, state, and municipal stakeholders for decision making on different managerial and funding aspects. The country has adopted a model of monitoring and evaluation (M&E) linked to the guidelines of the National Health Plan (NHP) to support the implementation of priority health policies [3].

Serious efforts have been made to improve the coverage and quality of the national health databases, including the intensive use of information technologies [4]. The expanded access and improved quality and coverage of these databases have resulted in their increased use, including for academic research [5]–[10].

In order to analyze the evolution of selected indicators during the final years in this study we adopted a specific framework (see Text S1) that included social determinants of health and risk factors, access to the three levels of the health system (primary, secondary, and tertiary), relevant health outcomes, private insurance, and household expenditures.

## Progress towards UHC in Brazil

### 

#### Social determinants of health and risk factors

There has been a large improvement in important health determinants over the past 25 years, with the major changes occurring in less developed municipalities. Between 1991 and 2010, poverty and illiteracy rates decreased significantly, while access to water, electricity, and sanitation have increased, with an observed general reduction of inequalities between municipalities. In the same period smoking prevalence has decreased, while obesity has increased in the Brazilian population.

#### Primary health care

**Figure 1:**
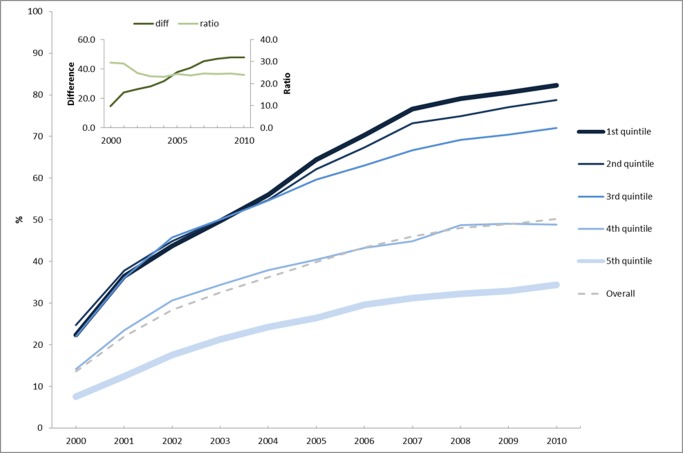
Average family health program coverage according to the municipal human development index (HDI-M) quintiles of the 5,507 Brazilian municipalities (data from the Primary Care Information System - SIAB [11]). The graph inset reflects the absolute differences and ratios between the FHP coverage of the poorest and richest quintiles.

All primary health care (PHC)-related activities have improved in Brazil in the last decade, with coverage of the chief strategy, the Family Health Programme (FHP), greatly increasing and reaching more than 50% of the population. The increase was larger in less developed municipalities, with low coverage maintained in the more developed municipalities (Figure 1).

#### Secondary health care

The percentage of hospital births in Brazil reached 91.8% in 2011, with the largest increase among less developed municipalities. The number of cesarean deliveries also increased, and the inequality measures among the selected indicators suggest a slight, and sometimes mixed, reduction in inequalities in secondary care.

#### Tertiary health care

Rates of hospital admission for cardiovascular surgery increased slightly from 2008 to 2011, but the large differences between more and less developed municipalities remained unchanged. Similar trends were observed in hemodialysis and the number of kidney transplants, suggesting a global maintenance of inequality in tertiary care.

#### Private insurance and health expenditures

The percentage of individuals ranging in age from 30 to 59 years who have private health insurance is close to 30%, reaching over 50% among wealthier individuals. Health expenditures in relation to the capacity to pay are higher but decreasing in the poorest quintiles of the population, and catastrophic health expenditures are minimal but still present, particularly in middle-income households.

#### Health outcomes

**Figure 2:**
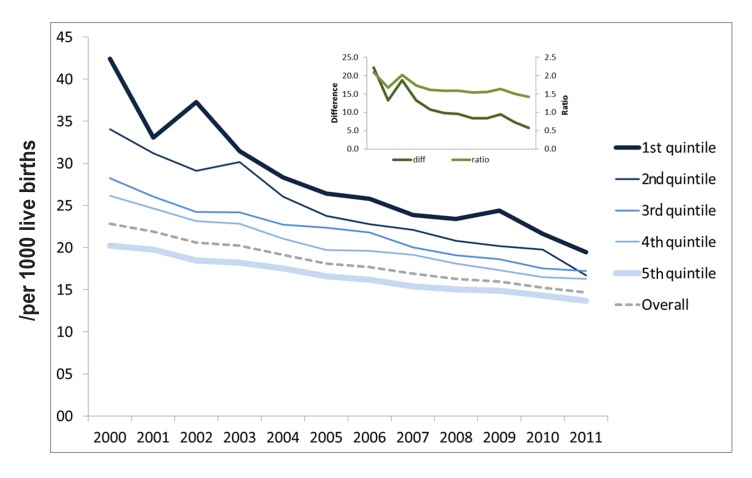
Under-five mortality rates according to municipal human development index (HDI-M) quintiles of the 5,507 Brazilian municipalities (Data from the Mortality Information System – SIM [12]). The graph inset reflects the absolute differences and ratios between the U5MRs of the poorest and richest quintiles.

Under-five mortality has greatly decreased in recent years, mainly in less developed municipalities, thereby reducing inequalities (Figure 2). There were significant increases in the percentage of individuals who reported a diagnosis of hypertension or diabetes, suggesting increasing access to health care.

## Conclusions and Recommendations

The SUS has guaranteed access to free health care for the population over the last 25 years. Overall, UHC has increased at all levels of care, with some important positive trends toward equity.

PHC, in particular through the expansion of the FHP, has succeeded in guaranteeing equitable coverage. FHP has demonstrated a high effectiveness and a synergistic effect with the national conditional cash transfer program (BFP) [5]–[9]. The final outcome was a marked reduction of inequalities in PHC access and utilization [10]; however, inequalities persist in secondary and tertiary care.

The chronic underfunding of the system imposes serious limitations on the overall expansion of the SUS, particularly at the secondary and tertiary levels [1]. In addition to ensuring adequate and sustained funding for the SUS, initiatives require support to increase access in all levels of care, and to improve the management of health services. Finally, continued monitoring of UHC indicators is recommended, with the goal of subsidizing policies to promote greater equity in health care provision and in the decrease of health determinants and risks.

## Supporting Information

Text S1 **The full country case study for Brazil.**
(DOCX)

## Abbreviations

FHP: Family Health Programme
PHC: primary health care
SUS: Brazilian Unified Health System
UHC: universal health coverage
