# Supplementary material for: Monitoring and Evaluating Progress towards Universal Health Coverage in Brazil
Source: PLoS Med. 2014 Sep 22;11(9):e1001692. doi: 10.1371/journal.pmed.1001692 (PMC4171375; doi:10.1371/journal.pmed.1001692)
Supplement: Text S1 — The full country case study for Brazil. (DOCX) [file pmed.1001692.s001.docx]

**Full Case Study: Monitoring and evaluating progress towards universal health coverage in Brazil**

Mauricio L. Barreto^1,2^*, Davide Rasella^1,2^, Daiane B. Machado^1^, Rosana Aquino^1,2^, Diana Lima^1,2^, Leila P. Garcia^3^, Alexandra C. Boing^4^, Jackson Santos^1^, Juan Escalante^5^, Estela M. L. Aquino^1^, Claudia Travassos^6^

^1^Instituto de Saúde Coletiva, Federal University of Bahia, Salvador, Brazil

^2^National Institute in Science, Technology and Innovation in Health (CITECS), Salvador, Brazil

^3^Instituto de Pesquisa Econômica Aplicada, Brasília, Brazil

^4^Federal University of Santa Catarina, Floranopólis, Brazil

^5^Secretaria de Vigilância a Saúde, Ministry of Health, Brasília, Brazil

^6^Instituto de Comunicação e Informação Científica e Tecnológica em Saúde (ICICT) /FIOCRUZ, Rio de Janeiro, Brazil

* Corresponding Author

Email: [mauricio@ufba.br](mailto:mauricio@ufba.br)

**This paper is the full country case study to accompany the summary paper “Monitoring and evaluating progress towards universal health coverage in Brazil” that is part of the Universal Health Coverage Collection. Not commissioned; externally reviewed.**

**Abstract:** In 1988, Brazil created a Unified Health System (SUS) based on the constitutional principles of health as a citizen’s right and a state’s duty. During the last 25 years, the SUS has improved access to free health care for the population. However, approximately one-quarter of the population, among the richest in the country, are covered by private health plan. The aim of this study was to explore how far Brazil has advanced in terms of health care coverage and access based on a framework that integrates health determinants; primary, secondary, and tertiary levels of care; health outcomes; and costs for families, with a particular focus on equity. Using secondary data from several sources of information, at the aggregate and individual levels, we analyzed trends in health outcomes and coverage at different health care levels using the concentration index, ratios and differences between extreme social strata as measures of inequality. Our results show that the SUS succeeded in covering basic health needs of the population, mainly due to the expansion of primary health care through the Family Health Care Program, with nearly 100% of children receiving complete immunization and nearly all pregnant women accessing antenatal care. The overall effect of the SUS on the health outcomes investigated is positive, and remarkable decreases have been registered in childhood mortality, particularly that associated with poverty-related diseases. Health expenditures are higher but are decreasing in the poorest quintiles of the population, and catastrophic health expenditures are still present, even if limited, particularly in middle-income households. The results presented here and in other studies published elsewhere confirm the view that the SUS is overall expanding coverage and increasing access to all levels of care. Important positive trends toward equity observed at the primary care level were attenuated or not observed when the secondary and, especially, tertiary levels of care were considered.

**Summary Points:**

1. The Brazilian National Health System (SUS) has guaranteed access to free health care for the population over the last 25 years, but one-quarter of Brazilians (among the richest) are covered by private health plans.

2. The relative abundance of administrative and survey data, at the aggregate and individual levels, has allowed for the analysis of the evolution of a wide range of health determinants, health outcomes, and health coverage indicators during recent years. Using an integrated approach and following a theoretical framework, recent trends in several health outcomes and health coverage indicators using concentration indices, differences, and relative inequality measures were analyzed.

3. Primary health care, in particular through the expansion of the Family Health Program, succeeded in guaranteeing near universal coverage, in particular in immunizations and antenatal care. Secondary and tertiary care coverage is expanding however, while large social and geographical inequalities still persist.

4. Health expenditures are higher but decreasing in the poorest quintiles of the population, and catastrophic health expenditures are minimal but still present, particularly in middle-income households.

5. The overall effect of the SUS on the health outcomes investigated is positive, and remarkable decreases have been registered in childhood mortality, particularly that associated with poverty-related diseases.

6. The SUS is expanding coverage overall, increasing access to all levels of care, with some important positive trends toward equity; however, under-financing and inequalities, in particular in secondary and tertiary health care still persist.

**1. Background**

Universal and equitable health care utilization was part of the Brazilian Constitution of 1988. It has been an important objective of the Unified Health System (Sistema Único de Saúde - SUS), which is based on the constitutional principle of health as a citizen’s right and a state’s duty (Art 196 Brazilian Constitution 1988) [1]. Sub-constructional laws organized SUS founded in five basic principles: a) “universality”, by which all those who are Brazilian citizens by birth or naturalization, have an inherent right to health provided by the State, which has the responsibility for promoting the health, protect citizens against the risks to which they are exposed and to ensure assistance in case of any health need; b) “integrality” states that citizens must receive comprehensive care, with priority given to preventive activities, without prejudice to treatment services; c) “equity” implicates that the health system should be able to promote actions for all citizens, and directed to decrease any social and regional inequalities in health; d) “decentralization” meaning that actions and health services integrate a regionalized and hierarchical network; and e) “social participation”, permanent councils and periodical conferences will guarantee the social control of SUS. According to these principles, universal health coverage (UHC) in Brazil is defined as the provision by the State of a set of actions aiming to provide comprehensive, universal preventive and curative care, according to anyone’s needs, through decentralised management and provision of health services, and promotes community participation.

Brazil is a large, heteregeneous country where, after two decades of military dictatorship (1964-1985), a democratic government was reestablished. In the past three decades of democracy, the country has undergone rapid economic and social changes, including changes in major social determinants of health and in the organization of the health system [1,2,3,4,5,6,7,8,9]. The political structure in Brazil consists of three levels of government with a great deal of autonomy (the federal government, 26 states and a federal district, and 5,570 municipalities). The country has an area of 8.5 million square kilometers and a population of slightly more than 200 million inhabitants. The population is under significant transition, becoming increasingly older (10.8% above 60 years old in 2010) and living in large cities (84.4% of the population lived in urban areas in 2010). The birth rate decreased from 6.3 to 1.8 births per woman from 1960 to 2006. Brazil is a high middle-income country with a gross domestic product (GDP) of US$ 2.25 trillion and a gross national income per capita of US$ 11,630 in 2012 [6]. It is a diverse country, presenting high levels of geographic, social, and ethnic inequalities. Despite reductions observed in the last decade, income inequality is still very high in the country (the Gini Index dropped from 0.594 in 2001 to 0.527 in 2011) [8]. Living conditions have also changed substantially [8,9].

Economic changes and social policies in the past two decades, targeted at decreasing poverty, have led to important changes throughout the country. This decrease is related to increases in the minimum wage and literacy, as well as the development of a structured conditional cash transfer program (Bolsa Família Program-BFP). Particularly significant impacts on reduced poverty and childhood nutrition and mortality have been observed. Changes in the Human Development Index estimated at the municipal level (HDI-M) have been impressive. In 1990, 99.2% of municipalities had an HDI-M below 0.600, and none of them had an HDI-M of 0.700 or above; in 2010, these values were 34.6% and 25.6%, respectively [9].

The SUS has improved access to health care in general, with particular improvement in areas such as primary care and emergency services. Additionally, the SUS has invested heavily in the expansion of human resources and technology, including major efforts to manufacture pharmaceutical and biotechnological products that are essential to the country. From the perspective of the right to health care, nearly all of the individuals who seek the SUS are treated, but the SUS is only partially used by the wealthiest portion of the population, especially for more expensive procedures. Approximately one-quarter of the Brazilian population, among the richest in the country, are covered by private health plans [1].

The Family Health Program (FHP) has been the main SUS primary health care (PHC) strategy, aiming to create a solid base for the health care system. Regarding secondary and tertiary SUS health services, despite efforts to expand access, they continue to be underfunded, with large limits on access [1]. Even with the SUS offering free health care services, in 2002-2003, health expenditures (including health insurance, medicines, and services) reached 7.2% of overall household expenditures [7], and catastrophic health expenditures reached approximately 2%, while varies depending on definitions [10]. The SUS resulted in important improvements in the Brazilian population’s health. However, because of certain structural factors (i.e., funding, shortages of trained health professionals) [1], availability of and access to services are not equitable at all levels of care; care does not reach everyone to the same extent, which generates equity concerns.

This study aimed to explore the extent to which Brazil has advanced in terms of health care coverage and utilization by considering a set of indicators presented in a framework (Figure S3) that integrates health determinants; primary, secondary, and tertiary levels of health care; health outcomes; and costs, with a particular focus on equity. A secondary aim was to contribute to the development and selection of a set of indicators with which to monitor health care coverage and equity at an international level.


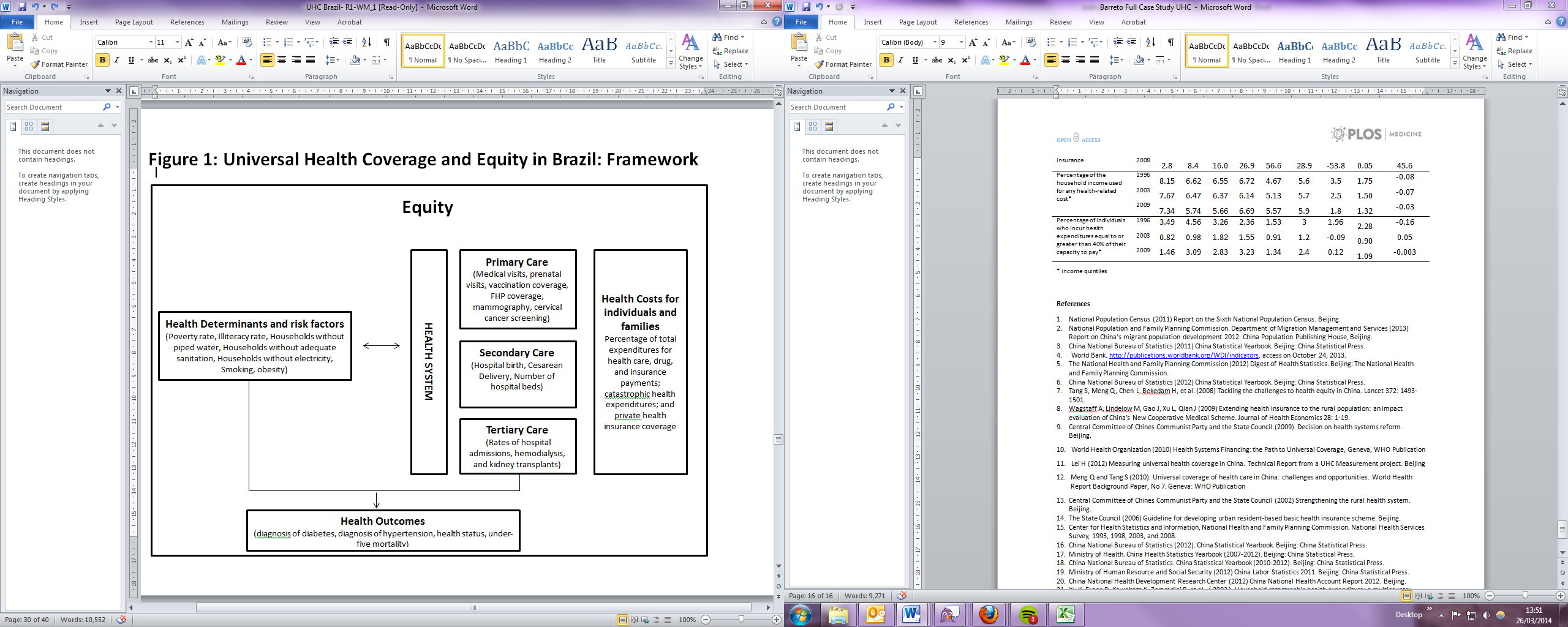


**Figure S3:** Universal Health Coverage and Equity in Brazil: Framework

**2. Universal health coverage: the policy context**

Since 1988, Brazil has been making efforts to develop a universal health system (Unified Health System-SUS) founded on the established constitutional principle. The management of this large and complex health system is conducted by a combination of mechanisms including executive bodies (at the federal, state, and municipal levels) and inter-managerial committees between the three levels of government, in addition to bodies organized to catalyze the social participation of the population and the numerous stakeholders involved (national, state, and municipal health councils). The Brazilian health system includes an intricate public-private mix. The SUS is financed by taxes and social contributions, with care provided by public or private services. Private insurance companies sell insurance for employers and individuals but are partially subsidized by the government through the fiscal mechanism of tax waivers. In 2007, Brazil spent 8.4% of the gross domestic product in health care, with the public share (federal, state and municipalities all together) 41% of the overall spending [1].

The SUS aims to provide comprehensive, universal preventive and curative care through decentralized management and provision of health services at the primary, secondary, and tertiary levels, whether directly or through the private sector, and promotes community participation.

Beyond health care, SUS has a fundamental role for the public health surveillance throughout the country [11]. This role is fulfilled by a large network of health surveillance units in 27 States and a major part of the 5,570 municipalities headed by the National Ministry of Health, together with the ANVISA (Agencia Nacional de Vigilância Santária), the National regulatory authority for food, drugs, services and environment. SUS also includes efforts to strengthen the training of health professionals at all levels, to define and fund a health research and development agenda based on national priorities, and to produce, either directly or with partners, the country’s most essential pharmaceutical and immune-biological products. Although it was not our objective here to analyze the contribution of SUS to these areas, it is important to mention that the understanding of the universal and equitable function of the Brazilian SUS mean that beyond direct actions on health prevention and care it needs to have complementary actions in several different fields aiming the strengthen the health system[11].

Despite the high priority of the FHP at all levels of government, FHP coverage has stabilized at approximately 55% of the Brazilian population, due to structural factors that imposes barriers to its expansion. Evaluative studies have consistently shown that the use of FHP services has been effective, improving important health outcomes [12,13,14,15,16].

In general, the health of Brazilian people is improving [2,3,4,5,6]. The pattern of mortality since the 1970s has been a predominance of non-communicable diseases (NCDs) (in particular, cardiovascular disease and cancers), whereas the incidence of infectious diseases has decreased sharply [3,4]. The current trends in the pattern of deaths due to infectious diseases (IDs) show pronounced decreases in general, particularly among children. The distribution of ID-related causes of death has shifted toward a pattern commonly seen in high-income countries, with a predominance of pneumonia in the elderly population. Other IDs have shown important decreases in incidence or prevalence; for instance, tuberculosis incidence has slowly but steadily decreased over the past 10 years, reaching a level below 40 per 100,000 inhabitants. These changes do not mean that older or newly emerged problems remain unsolved. Diseases such as leishmaniasis continue to slowly but steadily spread over new areas, and new dengue epidemics are always a possibility in urban centers throughout the country [3]. The overall rate of NCD mortality, in particular from cardiovascular diseases(CVD), has decreased since the 1990s, and overall cancer mortality is stable but has large variations when specific causes are considered; the mortality rates for some cancers are decreasing, but those for other cancers are increasing [4].

**3. Monitoring and evaluation for UHC**

In Brazil, the SUS has advanced in decentralized management processes, involving instances and negotiation mechanisms between federal, state, and municipal stakeholders for decision-making on different managerial and funding aspects. The country has adopted a model of monitoring and evaluation (M&E) linked to the guidelines of the National Health Plan (NHP) to support the implementation of priority health policies.

The M&E framework is based on the Pact for Health, a set of institutional reforms agreed upon between the three levels of government (federal, state, and municipality) of the National Health System, with the aim of promoting innovations in processes and management tools [17]. It includes a list of indicators for monitoring the annually defined goals to be achieved by each municipality or state.

Brazil has made serious efforts to improve the coverage and quality of the national health databases, described in Box S1, including the intensive use of information technologies. A considerable amount of these data are accessible (anonymously, and respecting all principles of privacy) in a central portal, DATASUS [18]. The expanded access and improved quality and coverage of these databases have resulted in their increased use, such as for academic research.

For this study, we adopted a specific framework (Figure S3) that included social determinants of health and risk factors, access to the three levels of the health system (primary, secondary, and tertiary), some relevant health outcomes, private insurance, and household costs. The method of analysis and the manner in which equity was approached are presented in Box S2, and a description of the inequality measures used is presented in Box S3.

| **Box S1: Brazilian health information systems and periodic health surveys**  The Department of Information Technologies of the SUS (DATASUS) is an agency of the Ministry of Health, the purpose of which is to gather, standardize, process, and disseminate information on health in Brazil. It is a center of technical support for health information dissemination, with centers at the regional and state levels. The DATASUS develops the national information systems and information technologies, maintains and manages national databases, disseminates data on the internet, and trains the staff responsible for processing health information [18].  The major health information systems (HIS) nationwide are:  MORTALITY INFORMATION SYSTEM (SIM): The oldest HIS in Brazil, created in 1975. Data are collected from death certificates and are standardized nationally. The record of cause of death is based on the 10th Revision of the International Classification of Diseases.  LIVE BIRTHS INFORMATION SYSTEM (SINASC): Introduced in 1990, provides information (standardized nationally) on live births in the country, with data on pregnancy, delivery, and the conditions at birth, from certificates of live birth.  NOTIFIABLE DISEASES INFORMATION SYSTEM (SINAN): Created in 1993 with the aim to collect, transmit, and disseminate surveillance data to support research on and analysis of mandatory-notification diseases using data derived from individual notifications and records research.  AMBULATORY-CARE INFORMATION SYSTEM (SIA): Created in 1992 based on the Outpatient Production Bulletin and includes information on the number of visits made to the SUS by type of procedure and population group. Information on highly complex procedures (e.g., hemodialysis and cancer therapy) that is recorded individually and detailed in the document serves as the basic authorization procedures for high cost/complexity (APAC).  HOSPITAL INFORMATION SYSTEM (SIH): Originally conceived in 1981 and extended in 1991 for all hospitalizations that are partly or fully paid by the SUS, which corresponds to 60-70% of the overall hospital admissions in the country, it varies according to region. Based on the Authorization for Hospitalization (AIH) document, the data it contains (admission diagnosis, procedures performed, and amounts paid) are individualized based on the patient and hospital.  NATIONAL IMMUNIZATION INFORMATION SYSTEM (SI PNI): Developed to guide the actions of the National Immunization Program (NIP), data collected refer to the number of individuals vaccinated at health facilities, the handling of biological (stock, distribution, use, technical, and physical losses) products and the reporting of adverse events.  NATIONAL REGISTER OF HEALTH ESTABLISHMENTS (CNES): Implemented nationally in 2003, this register provides information on the characteristics of establishments and health professionals, thus allowing a broad view of the physical and human resources existing in Brazil.  PRIMARY CARE INFORMATION SYSTEM (SIAB): Implemented in 1998, it collects information about housing and sanitation, health status, and composition of the families under the care of the health teams. It is the main instrument for monitoring the actions of the Family Health Program.  Several periodic representative surveys collect data on socioeconomic and demographic characteristics, risk factors and behaviors, health care facilities, and self-reported diseases.  The main periodic health and socioeconomic surveys/censuses are the following:  DEMOGRAPHIC CENSUS - IBGE: Held every 10 years since 1940, this census aims to obtain information on the population and households in the country, as well as to investigate a wide range of demographic and socioeconomic characteristics (gender, age, religion, color, race, nationality and national origin household characteristics, education, labor) and information on mortality, fertility, and migration.  NATIONAL HOUSEHOLDS SAMPLE SURVEY (PNAD) - IBGE: Conducted in a sample of Brazilian households annually since 1967. Investigates various socioeconomic characteristics (e.g., population, education, labor, income, housing, security, migration, fertility, marriage, health, and nutrition). It periodically produces supplements on specific topics such as health, food security, labor, and welfare. Of special interest with respect to health supplements are the 1998, 2003, and 2008 results on access to and utilization of health services. PNAD data can be analyzed at the country, region, federation unit, and major metropolitan levels.  HEALTH-MEDICAL ASSISTANCE (AMS) - IBGE: Applied annually since 1931, it collects information on health facilities (characterization, available services, production services, human resources, and equipment). It consists of census research, through interviews, that covers all health facilities in the country to assist with individual and collective health, whether outpatient or inpatient and including diagnosis and therapy.  HOUSEHOLD EXPENDITURE SURVEY (POF): Conducted since 1995, this is a household sample survey that register in detail the structure of the population’s expenditures and savings. Of particular importance to health was a survey on household consumption in terms of food, beverages, medications, and health plans that conducted anthropometric measurements of children and adolescents up to 19 years of age. The information in the POF can be detailed by region, state, class and income, and urban or rural residence.  RISK FACTORS SURVEILLANCE FOR NON-COMMUNICABLE DISEASES PREVENTION BY TELEPHONE SURVEY (VIGITEL): Conducted annually since 2006, it is focused on adults aged 18 or more years who live in the capitals of the 26 Brazilian states and the Federal District and in households with at least one fixed telephone line. The coverage of fixed telephone lines in the cities studied is approximately 70%, and factors are used to partially correct the bias resulting from low coverage and distributions of gender, age, and education.  NATIONAL SURVEY OF SCHOOL HEALTH (PeNSE): Collects data on nutritional and health behaviors, smoking, and physical activity, including family and school relationships, while measuring the weight and height of children in all Brazilian state capitals.  DEMOGRAPHIC HEALTH SURVEY (DHS). Conducted every 10 years, it is a comprehensive, nationally representative household survey, with a structure almost identical to that of other DHSs conducted world-wide. It covers population, education, health, nutrition, family planning, and household characteristics.  NATIONAL HEALTH SURVEY (PNS). The first Brazilian national health survey, scheduled for 2013. It includes clinical parameters and laboratory measurements. |
| --- |

| **Box S2: Data analysis methods**  Data source  Routinely collected data at the aggregate level were made available through the DATASUS (Department of Informatics of SUS) portal [18] from the following information systems: the Mortality Information System – SIM [19]; the Information System on Live Births – SINASC [20]; the Notifiable Diseases Information System – SINAN [21]; the Primary Care Information System – SIAB [22]; the Outpatient Information System – SIA [23]; and the Hospital Information System – SIH [24]. For socioeconomic and demographic variables, data were collected from the Brazilian Institute of Geography and Statistics (IBGE). Data at the individual level were collected from the Health Supplement of National Household Sample Surveys (PNAD) [25] for the years 1998, 2003, and 2008; the Family Expenditure Surveys (POF) [26] for the years 1996, 2003, and 2009; and the Surveillance System of Risk and Protective Factors for Chronic Non-Communicable Diseases through Telephone Interviews (VIGITEL) [27] for the years 2006-2011.  Level of Care and Indicators  Primary, secondary, and tertiary care were defined as follows [28]. Primary care is basic or general health care focused on the point at which a patient first seeks assistance from the medical care system. It is the basis for referrals to secondary- and tertiary-level care. Secondary care is specialist care provided on an ambulatory or inpatient basis, usually following a referral from a primary care center. Birth delivery, because it predominantly takes place in Brazil in the hospital with the assistance of doctors, was classified as secondary care. Tertiary care is the provision of highly specialized services in ambulatory and hospital settings.  The indicators used in the analysis were: Poverty rate, defined as the percentage of individuals with an income per capita lower than 60.9US$ per month; Illiteracy rate, defined as the percentage of individuals with 15 years or more who are not able to read or write a simple message; Household without piped water, defined as the percentage of households without a piped water in at least one of their rooms; Household without adequate sanitation, defined as the percentage of households with inadequate water supply and/or sewage; Household without electricity, defined as the percentage of households without any electric lighting; Percentage of smokers, defined as the percentage of individuals older than 18 years who declare to smoke, independently from the frequency or quantity; Percentage of obese individuals, defined as the percentage of individuals older than 18 years with a BMI>=30 according to auto-declared measures of high and weight; Immunization coverage of Tetravalent Vaccination, defined as the percentage of children younger than 1 year fully vaccinated for the combined immunization of diphtheria, tetanus, pertussis and Hib; Percentage of pregnant women without any prenatal visit at the moment of delivery; Percentage of pregnant women with 7 or more prenatal visits at the moment of delivery; Average coverage of the Family Health Program, defined as the number of individuals under a FHP catchment area divided by the total number of inhabitants; Smear-positive tuberculosis cure-rate, defined as the number of smear-positive patients cured; Percentage of deliveries at the hospital; Percentage of cesarean deliveries; Number of hospital beds - including public, private and nonprofit - per 1000 inhabitants; Rate of admission to hospital (per 100,000 inhabitants) for cardiovascular surgery; Number of hemodialysis done in a year per 1,000 inhabitants; Number of kidney transplantation per 100,000 inhabitants; Percentage of individuals 30-59 years old who underwent medical visits in the past 12 months; Percentage of women older than 25 years who underwent mammography at least once in life; Percentage of women between 25 and 59 years of age who underwent cervical cancer screening at least once in life, proportion of individuals 30-59 years old admitted to hospital in the past 12 months; Percentage of individuals from 30 to 59 years which self-assessment of health status was very good or good; Percentage of individuals from 30 to 59 years who reported physician-diagnosed hypertension; Percentage of individuals from 30 to 59 years who reported physician-diagnosed diabetes; Percentage of individuals from 30 to 59 years who have private health insurance; Percentage of the household income used for any health-related cost; Percentage of individuals who incur in a catastrophic health expenditure, defined as health expenditure equal or higher than 40% of their capacity to pay. |
| --- |

| **Box S3: Measuring inequalities**  The approach used the rank socio-economic strata was different for routine data available at aggregate level (municipalities) and surveys with data available at individual level. For data available only at municipality level, the following procedures were followed to classify municipalities in socio-economic levels: municipalities were stratified according to their Municipal Human Development Index (HDI-M) quintiles at the baseline year of 2000 (see Figure S6) [29]. Indicators under study were calculated for each quintile as the ratios between the sums of the outcome in the municipalities divided by the sum of the population. For each indicator, quintile trends were represented in graphs. This analysis allowed us to show not only how the average poverty level of the municipality can affect health service utilization and outcomes but also, in the case of health services available only in central urban municipalities (e.g., capitals), how the same aspects could be affected by other barriers to access (geographical, cultural, economic). For survey data available at the individual level, in each survey, subjects were stratified by income (five strata) or, if income was not available, by schooling (three strata). For each indicator, stratum trends were represented in graphs. This analysis allowed us to show how individual income or schooling level could affect the outcome under study, independent from the place of residence of the subjects (e.g., rural or urban).  Three different measures of inequality were used: the differences and ratios between the poorest and richest quintiles and the concentration index [30]. Whereas the first and second measures evaluate inequality using only the extreme quintiles of the distribution, the concentration index (CI) uses all quintiles to obtain a value that represents greater inequality the farther it is from 0, whether positive or negative, with +1 and -1 as the extreme values. The CI has a negative value when the indicator is concentrated among the poor, and it has a positive value when the health indicator is concentrated among the rich. To calculate the CI, a written program for STATA v12 was used [31]. Inequality was measured for all indicators shown in Tables S1-S5.  Regarding the first two measures, it has to be considered that in our Table S1, even though the difference in absolute numbers between the first and fifth quintiles is always dramatically decreasing, the ratio is often increasing. This phenomenon occurs because small reductions during the period in terms of absolute numbers in the fifth quintile (the richest) could represent large reductions in terms of percentage and could strongly influence the ratio for the first quintile, which possibly decreased much more in terms of absolute numbers but not in terms of percentage. For example, in the case of the absence of piped water, even if the reduction from 1991-2010 was, in absolute terms, more than 50 points in the first quintile (the poorest one), corresponding to 63% of the initial value, it was less than the 75% reduction registered in the fifth quintile (the richest one), due only to a6 points decrease in the same time period. A broad and critical evaluation of the values of each of the three measures of inequality, among others available, is necessary to judge whether reduced inequality has occurred for a certain indicator.  **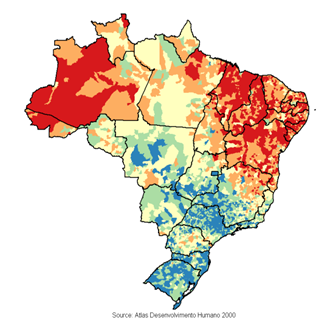**  **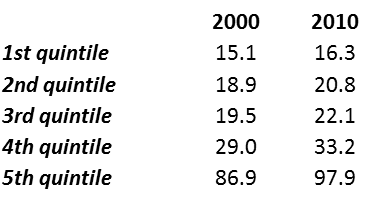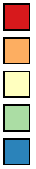 Total population (in millions) of each HDI-M quintile, 2000-2010**  **Figure S6:** Map of Brazilian muncipalities classified according to the 2000 Municipal Human Development Index quintiles.  Data Source: [29] |
| --- |

**4. Progress towards UHC in Brazil**

**4.1 Health Determinants and Risk Factors**

Table S1 shows a large improvement in all health determinants in the period under study, with the major changes, in terms of absolute values, taking place in the municipalities with the lowest HDI-M (first quintile). In the period 1991-2010, the poverty rate decreased from 82.1 to 48.0 in the lower quintile, and the difference between the first and fifth quintiles decreased from 64.4 to 42.6. Following the same trend, the difference in illiteracy rates between the first and fifth quintiles decreased from 44.4 to 26.0. Concentration indexes slightly decreased in both cases. As discussed above, this reduction of socioeconomic inequalities has been strong enough for the last decade in the country to be called the "inclusive decade" [8,32]. The number of households without piped water also decreased the most in the first quintile; the difference between the municipalities in the two extreme quintiles changed from 72.5 to 27.6. The difference in the percentage of households without adequate sanitation between the first and fifth quintiles decreased from 44.5 to 22.9, and the ratio decreased from 36.2 to 28.2; the concentration index also slightly improved. The difference in the percentage of households without an electrical power supply also decreased between these two groups from 52.8 to 6.1%. The increases in water supply and electricity over the last decade were boosted by specific federal programs, such as Water-for-All and Electricity-for-All [33,34].

Regarding smoking (Table S2), in both genders, individuals with fewer than eight years of schooling smoked the most; however, this percentage is slightly decreasing among men from 24.2% in 2006 to 22.3% in 2010. Among women, the smoking prevalence is lower and the tendency is stable. These results confirm the findings from other studies that demonstrate that lower schooling levels were associated with a higher prevalence of smoking and that the prevalence of smoking declined substantially in Brazil, from 34.8% in 1989 to 17.2% in 2009, likely due to the broad framework of laws implemented to control tobacco use [35,36]. The percentage of obese individuals with all levels of education is increasing, but the highest increase is in women with fewer than 8 years of schooling, reaching 20.3% in 2010. The immediate determinants of this increase are unknown, but specific policies have been implemented in recent years to promote physical activity and reduce the consumption of unhealthy foods [4].

| Year | HDI-M quintiles | | | | |  | Diff | Ratio | Concentration index |
| --- | --- | --- | --- | --- | --- | --- | --- | --- | --- |
|  | 1st | 2nd | 3rd | 4th | 5th | Overall | 1^st^ – 5^th^ | 1^st^/5^th^ | 1^st^ to 5^th^ |
| Poverty rate | | | | | | | | |  |
| 1991 | 82.1 | 73.6 | 56.4 | 37.1 | 17.7 | 38.1 | 64.4 | 4.6 | -0.25 |
| 2000 | 69.9 | 59.1 | 39.5 | 24.6 | 12.3 | 27.9 | 57.6 | 5.7 | -0.29 |
| 2010 | 48 | 37 | 20.4 | 10.9 | 5.4 | 15.2 | 42.6 | 8.9 | -0.37 |
| Illiteracy rate | | | | | | | | |  |
| 1991 | 53.8 | 41.9 | 26.4 | 16.2 | 9.4 | 20.5 | 44.4 | 5.8 | -0.31 |
| 2000 | 38.6 | 28.7 | 17.4 | 10.5 | 6 | 13.5 | 32.4 | 6.4 | -0.32 |
| 2010 | 30.2 | 22.2 | 12.9 | 7.5 | 4.2 | 10 | 26 | 7.2 | -0.35 |
| Households without piped water | | | | | | | | |  |
| 1991 | 80.5 | 68.1 | 44.2 | 22.7 | 8 | 28.6 | 72.5 | 10.1 | -0.34 |
| 2000 | 68.2 | 53.2 | 29.2 | 14.9 | 4.8 | 20.4 | 63.4 | 14.2 | -0.39 |
| 2010 | 29.6 | 20.2 | 9.8 | 5.9 | 2 | 7.9 | 27.6 | 14.8 | -0.41 |
| Households without adequate sanitation | | | | | | | | |  |
| 1991 | 45.8 | 31.1 | 10.3 | 3 | 1.3 | 10.3 | 44.5 | 36.2 | -0.51 |
| 2000 | 23.9 | 24.7 | 15.6 | 7.7 | 1.8 | 8.9 | 22.1 | 13.2 | -0.33 |
| 2010 | 23.7 | 18.2 | 8.4 | 4 | 0.8 | 6.1 | 22.9 | 28.2 | -0.44 |
| Households without electricity | | | | | | | | |  |
| 1991 | 54.5 | 42 | 23 | 8.7 | 1.7 | 15.1 | 52.8 | 32 | -0.43 |
| 2000 | 28.9 | 20.8 | 8.8 | 2.4 | 0.4 | 6.5 | 28.5 | 68.5 | -0.49 |
| 2010 | 6.2 | 4.7 | 1.9 | 0.5 | 0.1 | 1.4 | 6.1 | 52.6 | -0.49 |

**Table S1:** Means of the selected variables according to the municipal Human Development Index (HDI-M) quintiles, and values of the differences and ratios between the poorest and richest quintiles.

| Variable | Year | Years of schooling | | | | | | | |  |
| --- | --- | --- | --- | --- | --- | --- | --- | --- | --- | --- |
|  |  | <8 years | |  | 9-11 years | |  | >11 years | |  |
|  |  | Male | Female | Overall | Male | Female | Overall | Male | Female | Overall |
| Percentage of smokers | 2006 | 24.2 | 14.7 | 19.1 | 16.3 | 11.1 | 13.4 | 14.4 | 8.9 | 11.5 |
|  | 2010 | 22.3 | 15.5 | 18.6 | 13.6 | 9.9 | 11.6 | 11.5 | 9.1 | 10.2 |
| Percentage of obese individuals | 2006 | 12 | 15.2 | 13.6 | 9.9 | 8.1 | 8.9 | 11.8 | 7.5 | 9.6 |
|  | 2010 | 14.3 | 20.3 | 17.3 | 13.6 | 11.1 | 12.2 | 16.1 | 10.7 | 13.3 |

**Table S2**: Values of the selected variables according to years of schooling.

**4.2 Primary Health Care**

Primary care coverage and, consequently, all PHC-related activities have improved in Brazil in the last decade (Table S3). FHP coverage largely increased in the three poorest quintiles, with low coverage maintained in the richest municipalities (Figure S1). The FHP has been demonstrated to be effective during the last decade in increasing vaccination coverage and antenatal care [13], reducing avoidable hospital admissions [37,15,16], improving vital information, and reducing mortality rates in children under 5 years of age [14]. Moreover, the FHP has demonstrated a synergistic effect with poverty-reduction interventions such as the BFP [38,39], and it is contributing to the reduction of inequalities in health access and utilization [40]. Immunization coverage for the tetravalent vaccine (diphtheria, tetanus, pertussis, and Hib) was nearly 100% across the country during the study period. The achievement of near-universal coverage with measles and DPT3 immunizations is likely one of the factors that have contributed to the country’s reduced child mortality in the last 40 years since the creation of the National Immunization Program [41]. A recent field study confirmed the tendency for higher immunization coverage in low-income populations [42]. The percentage of pregnant women who had no prenatal visits prior to delivery decreased to less than 5% , with the highest decrease found in the first quintile (Figure S4); meanwhile the number of pregnant women with 7 or more prenatal visits increased in the first quintile to from 19.2 to 40.8% (Table S3). The health conditionalities of the BFP (which include prenatal care, postnatal care, and health and nutrition educational activities for mothers, in addition to a regular vaccination schedule and routine growth and development check-ups for children younger than 7 years) are among the broad range of factors that contributed to the increased immunization rates and prenatal visits [38]. However, it must be considered that despite the great improvement in recent years, antenatal care is still poorly integrated with delivery care [2]. The percentage of women between 25 and 59 years who underwent a cervical cancer screening or a mammography at least once in life increased slightly more among the lower-income group during the period 2003-2008 (Table S4), but the inequalities across income levels are still large, as confirmed in recent studies [43,44]. The increase in cervical cancer screening, which has been ongoing since the 1980s, has paralleled the decrease in deaths attributable to cervical cancer over the past two decades; however, large variations still exist across income levels [4]. Age-standardized mortality rates for breast cancer are increasing, and it is now the most common cause of death from cancer in women [44]. According to some estimates, only 30-35% of women aged 50-69 years receive appropriate breast screening, and 80% of women reported not having a doctor’s referral as the main barrier to screening. The percentage of individuals 30-59 years old who underwent doctor visits in the past 12 months increased, with a similar trend in all income per capita groups. In primary health care, nearly all measures of inequality investigated (difference, ratio, and CI) indicate an important reduction of inequality.


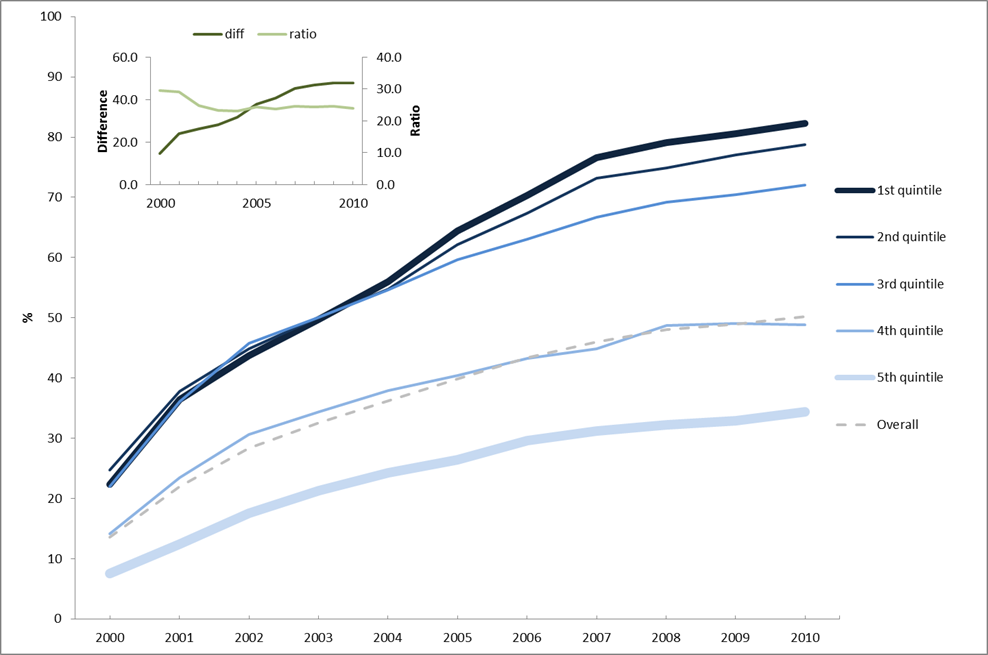


**Figure S1:** Average Family Health Program coverage according to the HDI-M quintiles of the 5507 Brazilian municipalities.

Data Source: Primary Care Information System(SIAB)

Note: The graph inset reflects the absolute differences and ratios between the indicator values of the poorest and richest quintiles.


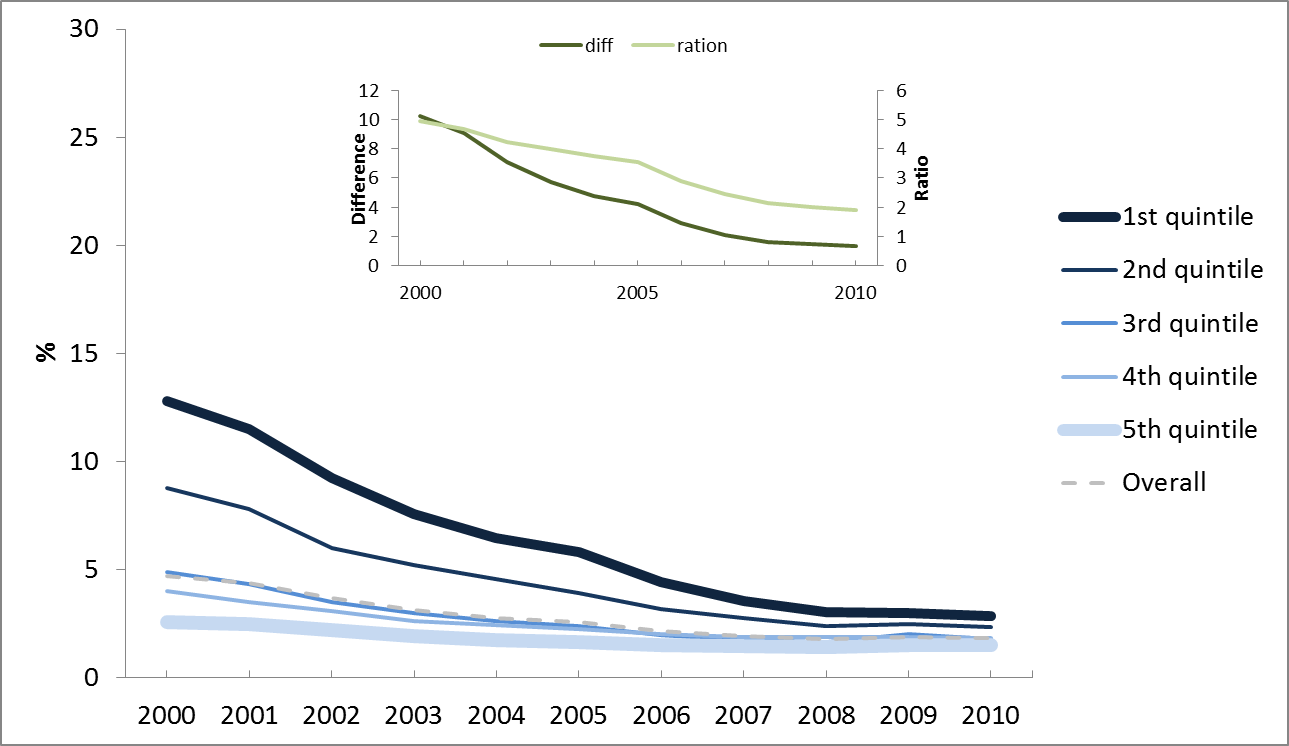


**Figure S4:** Trends in the percentage of pregnant women with no prenatal visits prior to delivery according to municipal HDI-M quintile.

Data Source: Life Births Information System(SINASC)

Note: The graph inset reflects the absolute differences and ratios between the indicator values of the poorest and richest quintiles.

| Variable | Year | HDI-M quintiles | | | | |  | Diff | Ratio | Concentration index |
| --- | --- | --- | --- | --- | --- | --- | --- | --- | --- | --- |
|  |  | 1st | 2nd | 3rd | 4th | 5th | Overall | 1^st^ – 5^th^ | 1^st^/5^th^ | 1^st^ to 5^th^ |
| Primary Health Care | | | | | | | | | |  |
| Imunization Coverage Tetravalent vaccination | 2003 | 108.7 | 109.8 | 110.9 | 101.9 | 97.3 | 102.5 | 11.4 | 1.1 | -0.02 |
|  | 2011 | 101.2 | 102.2 | 103.8 | 100.5 | 96.9 | 99.4 | 4.2 | 1.0 | -0.008 |
| Percentage of pregnant women without any prenatal visit at the moment of delivery | 2000 | 12.8 | 8.8 | 4.9 | 4.0 | 2.6 | 4.7 | 10.2 | 4.9 | -0.30 |
|  | 2011 | 4,8 | 3.8 | 2.7 | 3.0 | 1.9 | 2.7 | 2.9 | 2.5 | -0.16 |
| Percentage of pregnant women with 7 or more prenatal visits at the moment of delivery | 2000 | 19.2 | 23.1 | 35.1 | 43.0 | 54.9 | 50.4 | 35.7 | 0.4 | 0.21 |
|  | 2011 | 40.8 | 46.4 | 54.6 | 58.8 | 71.3 | 69.1 | 30.5 | 0.6 | 0.10 |
| Average coverage of the Family Health Program | 2000 | 22.4 | 24.7 | 22.0 | 14.2 | 7.5 | 13.6 | 14.8 | 3.0 | -0.18 |
|  | 2010 | 82.3 | 78.7 | 72.0 | 48.9 | 34.5 | 50.3 | 47.8 | 2.4 | -0.16 |
| Smear-positive tuberculosis cure-rate | 2001 | 81.5 | 86.2 | 83.6 | 83.7 | 77.5 | 80.3 | 4.0 | 1.1 | -0.01 |
|  | 2011 | 86.3 | 85.5 | 86.9 | 86.1 | 83.4 | 84.6 | 3.0 | 1.0 | -0.004 |
| Secondary Health Care | | | | | | | | | |  |
| Percentage of delivery at the hospital | 2000 | 82.4 | 86.2 | 89.7 | 94.2 | 91.2 | 90.3 | -8.8 | 0.9 | 0.02 |
|  | 2011 | 88.8 | 90.4 | 92.4 | 94.7 | 91.4 | 91.8 | -2.6 | 1.0 | 0.008 |
| Percentage of cesarean delivery | 2000 | 15.1 | 19.8 | 30.2 | 37.4 | 43.2 | 35.6 | -28.1 | 0.3 | 0.20 |
|  | 2011 | 32.4 | 37.9 | 47.0 | 53.4 | 56.2 | 50.3 | -23.8 | 0.6 | 0.11 |
| Number of hospital beds per 1000 inhabitants | 2005 | 1.5 | 2.1 | 2.3 | 2.5 | 2.8 | 2.5 | -1.3 | 0.5 | 0.11 |
|  | 2011 | 1.3 | 1.8 | 2.0 | 2.2 | 2.8 | 2.4 | -1.5 | 0.5 | 0.13 |
| Tertiary Health Care | | | | | | | | | |  |
| Rate of hospital admissions (per 100,000 inhab) for cardiovascular surgery | 2008 | 11.6 | 16.0 | 24.7 | 33.4 | 44.0 | 34.1 | -32.4 | 0.3 | 0.25 |
|  | 2011 | 14.8 | 20.0 | 29.1 | 39.0 | 48.5 | 38.6 | -33.8 | 0.3 | 0.31 |
| Number of hemodialysis per 1,000 inhabitants | 2008 | 22.4 | 29.9 | 42.4 | 58.1 | 63.3 | 52.8 | -40.9 | 0.4 | 0.20 |
|  | 2011 | 30.9 | 38.3 | 53.3 | 69.1 | 68.3 | 60.2 | -37.5 | 0.5 | 0.16 |
| Number of kidney transplants per 100,000 inhabitants | 2000 | 0.1 | 0.2 | 0.5 | 1.2 | 2.1 | 1.4 | -2.0 | 0.1 | 0.49 |
|  | 2011 | 0.5 | 0.9 | 1.5 | 2.0 | 3.2 | 2.3 | -2.7 | 0.2 | 0.32 |

**Table S3:** Values of the selected variables according to the municipal Human Development Index (HDI-M) quintile, and values of the differences and ratios between the poorest and richest quintiles

| Variable | Year | Income per Capita in Minimum Wages (MW) | | | | |  | Diff | Ratio | Concentration index |
| --- | --- | --- | --- | --- | --- | --- | --- | --- | --- | --- |
|  |  | <1 MW | 1-2 MW | 2-3 MW | 3-5 MW | >5 MW | Overall | 1^st^ – 5^th^ | 1^st^/5^th^ | 1^st^ to 5^th^ |
| Health Care | | | | | | | | | |  |
| Percentage of individuals 30-59 years old who underwent medical visits in the past 12 months | 1998 | 53.1 | 53.7 | 55.4 | 56.8 | 62.8 | 58.9 | -9.7 | 0.85 | 0.03 |
|  | 2003 | 59.5 | 60.5 | 63.1 | 65.6 | 72.1 | 66.0 | -12.6 | 0.83 | 0.04 |
|  | 2008 | 63.8 | 66.9 | 68.9 | 70.5 | 76.5 | 70.9 | -12.7 | 0.83 | 0.03 |
| Percentage of women older than 25 years who underwent mammography at least once in life | 2003 | 39,1* | 54.3 | 63.9 | 70.6 | 76.6 | 42.5 | -37.5 | 0.51 | 0.12 |
|  | 2008 | 47,7* | 61 | 71.1 | 76.3 | 81.1 | 54.5 | -33.4 | 0.59 | 0.10 |
| Percentage of women between 25 and 59 years of age who underwent cervical cancer screening at least once in life | 2003 | 83* | 88.8 | 92.1 | 94.6 | 96 | 82.6 | -13 | 0.86 | 0.03 |
|  | 2008 | 85,3* | 89.8 | 93.2 | 94.3 | 96.2 | 87 | -10.9 | 0.89 | 0.02 |
| Percentage of individuals 30-59 years old admitted to hospital in the past 12 months | 1998 | 9.7 | 8.7 | 7.7 | 6.9 | 6.6 | 7.3 | 3.1 | 1.47 | -0.08 |
|  | 2003 | 8.9 | 7.6 | 6.9 | 6.8 | 7.2 | 7.3 | 1.7 | 1.24 | -0.04 |
|  | 2008 | 8.4 | 7.1 | 7.3 | 7.0 | 7.1 | 7.2 | 1.3 | 1.19 | -0.03 |
| Health Outcomes | | | | | | | | | |  |
| Percentage of individuals from 30 to 59 years who self-reported good or very good health status | 1998 | 55.5 | 61.1 | 63.3 | 68.0 | 78.3 | 70.6 | -15.1 | 0.71 | 0.06 |
|  | 2003 | 58.2 | 63.2 | 66.8 | 71.0 | 81.0 | 71.3 | -13.1 | 0.72 | 0.06 |
|  | 2008 | 56.1 | 62.0 | 66.6 | 71.1 | 81.0 | 70.4 | -14.3 | 0.69 | 0.07 |
| Percentage of individuals from 30 to 59 years who reported physician-diagnosed hypertension | 1998 | 20.4 | 18.9 | 18.9 | 17.8 | 16.2 | 17.5 | 4.2 | 1.26 | -0.04 |
|  | 2003 | 20.6 | 19.5 | 19.4 | 19.1 | 17.4 | 18.8 | 3.2 | 1.19 | -0.03 |
|  | 2008 | 21.3 | 19.5 | 20.0 | 19.8 | 18.2 | 19.4 | 3.0 | 1.17 | -0.02 |
| Percentage of individuals from 30 to 59 years who reported physician-diagnosed diabetes | 1998 | 2.6 | 2.3 | 2.8 | 2.8 | 3.1 | 2.9 | -0.2 | 0.85 | 0.04 |
|  | 2003 | 3.3 | 3.2 | 3.3 | 3.6 | 3.6 | 3.4 | -0.2 | 0.91 | 0.02 |
|  | 2008 | 4.1 | 4.2 | 4.4 | 4.4 | 4.4 | 4.3 | -0.2 | 0.93 | 0.01 |
| * from 1/2 to 1 MW |  |  |  |  |  |  |  |  |  |  |

**Table S4:** Values of the selected variables according to income per capita, and values of the differences and ratios between the lowest and highest incomes

**4.3 Secondary Health Care**

The percentage of hospital deliveries in the country reached 91.8% in 2011 (Table S3). The largest increase occurred among municipalities in the first HDI-M quintile, increasing from 82.4% to 88.8% in the last decade. The number of cesarean deliveries also increased primarily in this group of municipalities, from 15.1% to 32.4% in the period from 2000-2011. A recent study showed that cesarean sections are more frequent among wealthier mothers, and this result was strongly related to maternal education among patients in the public sector [45]. The rate of hospital admissions in the last 12 months decreased in the lower-income groups and slightly increased in the higher income groups, demonstrating a reduction in the differences between them (Table S4). A possible explanation could be that the higher rates of hospital admissions reported by the low-income group were attributable to a higher frequency of severe disease episodes, with the subsequent need for healthcare, and to the use of hospitals as a substitute for primary care. The decrease was attributable to observed socioeconomic improvements, the effectiveness of primary health care, and supply changes within the SUS that occurred during the last period [14, 37, 40]. Inequality measures among the selected indicators suggest a slight, and sometimes mixed, reduction in inequalities in secondary care.

**4.4 Tertiary Health Care**

The rates of hospital admission for cardiovascular surgery increased slightly from 2008-2011, but the large difference between municipalities with lower and higher HDI-M values remained unchanged (Table S3). Socioeconomic factors have a great influence on the distribution, severity, and management of cardiovascular diseases in Brazil, with a higher mortality rate found in the low-income population because of differences in access to treatment and in exposure to risk factors [46,47]. Similar trends were observed in hemodialysis and the number of kidney transplants, in which the difference between the more- and less-developed municipalities has tended to increase, as shown in Figure S5. These findings confirm the results of a recent study that showed that patients residing in cities with higher HDI-Ms have more access to renal transplantation, likely because of better access to health care, better income and schooling, and not related with the transplant allocation system, which generates the waiting list, fair and of recognized transparency [48]. Inequality measures among the selected indicators suggest a mixed picture of slightly reduced or slightly increased inequalities, depending on the measure and the indicator, in tertiary care and of the global maintenance of inequality.


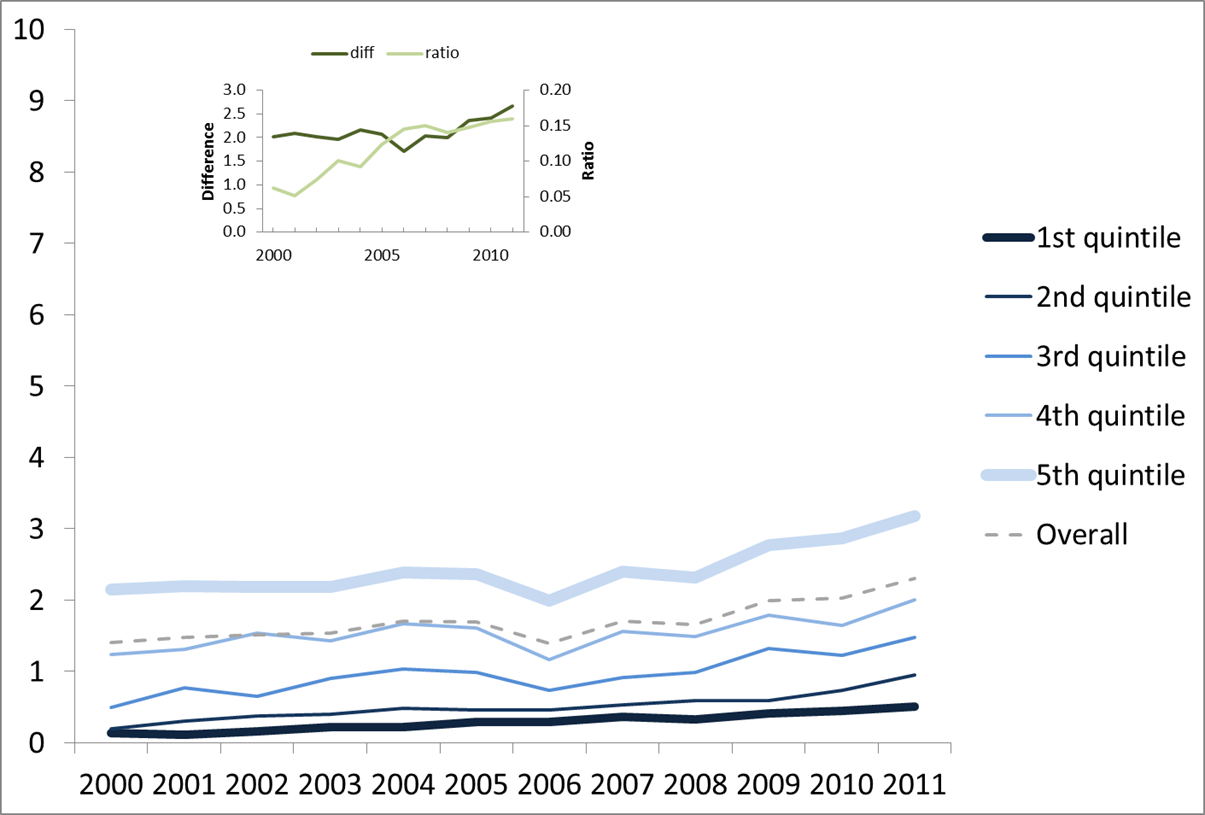


**Figure S5:** Trends in the number of kidney transplants per 100,000 inhabitants per year according to municipal HDI-M quintile.

Data Source: Hospital Information System(SIH)

Note: The graph inset reflects the absolute differences and ratios between the indicator values of the poorest and richest quintiles.

**4.5 Private Insurance and Health Expenditures**

Private health insurance coverage is highly dependent on income per capita and ranges from more than 50% among individuals who earn 5 or more times the minimum wage (MW) to below 3% among individuals with who earn less than the MW per capita, increasing among people who earn 1-2 times the MW or above. (Table S5). Health expenditures, intended as the percentage of household income used for any health-related cost, slightly decreased in the period from 1996-2009 among individuals in the three poorest income quintiles; nonetheless, the group with the lowest income per capita maintained the highest health expenditures (approximately 8%) during the entire period. Studies have shown that in lower-income families, the cost of medications represents the greatest portion of health expenditures, whereas in the richest families, great part of health expenditures go to private insurance fees [49]. Catastrophic health expenditures, defined as the percentage of individuals who incurred a health expenditure equal to or greater than 40% of their capacity to pay, decreased from 1996-2009, particularly in the poorest income quintile, reaching 1.49% in the year 2009 and approximately 3% in the middle-income group. While extremely poor families rely uniquely on public health providers and on free or low-cost drugs [50], middle-class families both with and without private insurance incur catastrophic health expenditures likely by seeking what they anticipate will be the faster or better care offered by private providers.

| Variable | Year | Income per Capita in Minimum Wages (MW) | | | | |  | Diff | Ratio | Concentration index |
| --- | --- | --- | --- | --- | --- | --- | --- | --- | --- | --- |
|  |  | <1 MW | 1-2 MW | 2-3 MW | 3-5 MW | >5 MW | Overall | 1^st^ – 5^th^ | 1^st^/5^th^ | 1^st^ to 5^th^ |
| Percentage of individuals from 30 to 59 years who have private health insurance | 1998 | 2.1 | 4.7 | 8.9 | 18.5 | 49.3 | 29.1 | -47.2 | 0.04 | 0.52 |
|  | 2003 | 2.1 | 6.3 | 13.4 | 24.4 | 55.7 | 28.6 | -53.6 | 0.04 | 0.49 |
|  | 2008 | 2.8 | 8.4 | 16.0 | 26.9 | 56.6 | 28.9 | -53.8 | 0.05 | 0.46 |
| Percentage of the household income used for any health-related cost* | 1996 | 8.15 | 6.62 | 6.55 | 6.72 | 4.67 | 5.6 | 3.5 | 1.75 | -0.08 |
|  | 2003 | 7.67 | 6.47 | 6.37 | 6.14 | 5.13 | 5.7 | 2.5 | 1.50 | -0.07 |
|  | 2009 | 7.34 | 5.74 | 5.66 | 6.69 | 5.57 | 5.9 | 1.8 | 1.32 | -0.03 |
| Percentage of individuals who incur health expenditures equal to or greater than 40% of their capacity to pay* | 1996 | 3.49 | 4.56 | 3.26 | 2.36 | 1.53 | 3 | 1.96 | 2.28 | -0.16 |
|  | 2003 | 0.82 | 0.98 | 1.82 | 1.55 | 0.91 | 1.2 | -0.09 | 0.90 | 0.05 |
|  | 2009 | 1.46 | 3.09 | 2.83 | 3.23 | 1.34 | 2.4 | 0.12 | 1.09 | -0.003 |
|  |  |  |  |  |  |  |  |  |  |  |
| * income quintiles |  |  |  |  |  |  |  |  |  |  |

**Table S5:** Values of the selected variables according to individual income per capita, and values of the differences and ratios between the lowest and highest incomes.

**4.6 Health Outcomes**

Under-five mortality has greatly decreased in recent years, mainly in municipalities with lower HDI-Ms (Figure S2), because of the reduced mortality associated with infectious poverty-related diseases, especially in the post-neonatal and 1-4-year-old age groups [2,41]. The percentage of individuals from 30-59 years of age who reported a diagnosis of hypertension increased overall from 17.5% in 1998 to 19.4% in 2008 (Table S4). The highest increase occurred among people who received more than 5 times the MW per month; however, this group still had the lowest percentage of individuals diagnosed with hypertension (18.2% in 2008). The percentage of individuals from 30-59 years of age who reported a diagnosis of diabetes increased among all classes during the period of study. The percentage of individuals who self-reported a good or very good health status in Brazil increased with income category; 81.0% of those who received more than 5 times the MW per month reported their health as good or very good in 2008, and 56.1% of those who received less than the MW had the same self-evaluation results (Table S2).

**
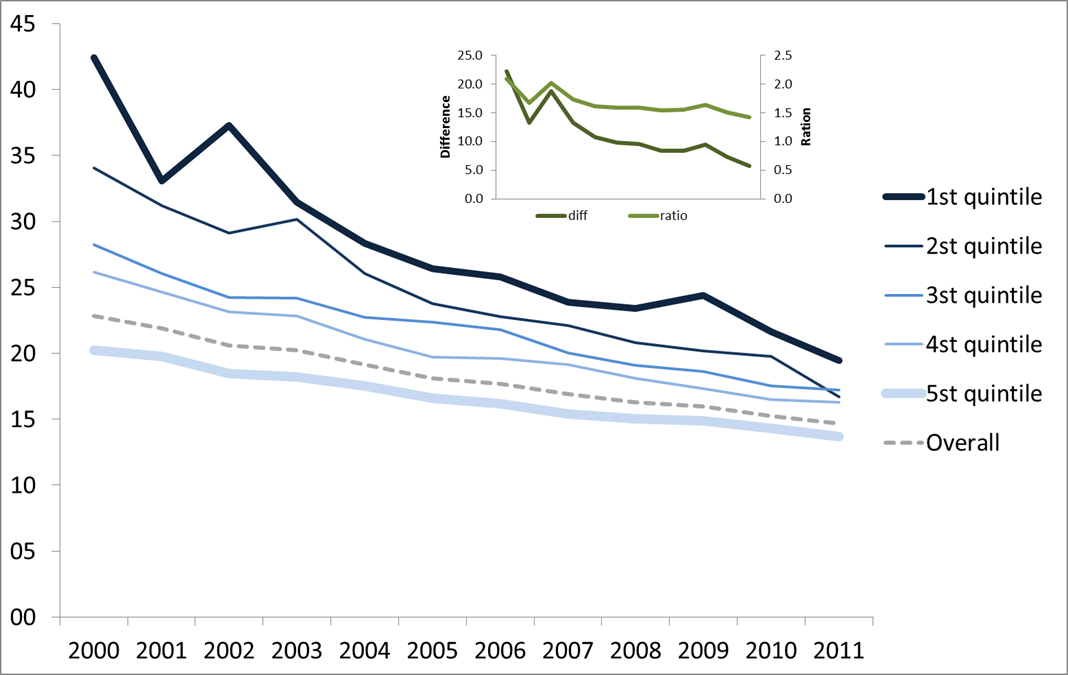
**

**Figure S2**: Under-five mortality rates according to HDI-M quintiles of the 5507 Brazilian municipalities.

Data Source: Mortality Information System(SIM)

Note: The graph inset reflects the absolute differences and ratios between the indicator values of the poorest and richest quintiles.

**5. Conclusions and recommendations (Box S4)**

In the course of the last two decades, important changes have been observed in Brazil regarding health determinants, health status, and access to health care [1,4,5,6,7]; however, several important challenges remain [51]. The expansion of the FHP, as the fundamental PHC strategy, is now achieving high coverage in small municipalities; together with the traditional PHC, the expansion is making PHC almost universal. However, questions persist related to problem-solving capacity, particularly for more complex clinical cases and to the PHC role in articulating the higher levels of care. Moreover, FHP expansion has been limited in the past few years among other possible causes the shortage of physicians. A special program was recently created (Federal Law Num. 12.871, 22nd October 2013) to boost medical training and encourage physicians from other countries to work in Brazil. Nearly 100% of children receive complete immunizations, which entails a large number of vaccines (BCG, DTP/HB/HiB, MMR, 10-valent pneumococcal, rotavirus), and access to antenatal care (including at least one medical consultation) among pregnant women is nearly universal.

The SUS covers the basic health needs of a substantial portion of the population, including distribution of medicines for a wide range of health problems (e.g., tuberculosis, HIV/AIDS, diabetes, hypertension, asthma, and psychiatric diseases); however, this group shares part of the health costs, and expenditures can eventually, in some cases, reach catastrophic levels. The overall effects on selected health outcomes are positive, and impressive decreases have been registered in childhood deaths, especially those from diarrhea, undernutrition, and pneumonia [2,41]. Infectious disease occurrence and mortality are generally decreasing, and CVD mortality has been decreasing since the 1990s [3,4]. When secondary and, in particular, tertiary care are considered, despite the efforts to increase access, important barriers are still present and important inequalities in access persist. The lack of access at the highest level of care is more marked in the lower-income quintiles.

Several initiatives that originated in the SUS to monitor and evaluate overall and specific aspects of the health system, including population health and user satisfaction with their healthcare, have been conducted, and a large number of technical groups dedicated to these tasks are spread throughout the different levels of the SUS managerial structure. Complementing these efforts, the SUS has dedicated financial investments to a large number of calls for academic groups to perform independent evaluative studies [52].

The availability of reliable data sources related to health determinants, health care, and outcomes is a potential advantage to health monitoring and evaluation in Brazil. The current policy of making a number of these databases accessible and free to potential users made possible a great number of evaluative initiatives that originated in academic groups, making this topic broader and not restricted to institutional initiatives within SUS management. The independent evaluative research resulted in greater overall SUS transparency and in a compromise in the academic community to contribute to the identification of gaps to be addressed, including suggestions on how to improve the accuracy and reliability of these databases. The quality of some of these databases has been confirmed by recent evaluative studies estimating the magnitude of the impact of health and social policies on mortality, hospital admission rates, and health care service utilization (37,38,39). A new generation of health information systems using the new possibilities created by the development of information technology is already being tested. The individual card for each SUS user participating in the pilot, which will be mandatory in each contact with the health system, shows great promise for integrating information and tracing individual users in the SUS [53].

Considering that equity, in its numerous dimensions, has been central to the principles of the SUS, the overall framework that guides the constitution of these health information systems has had a relatively weak focus on equity issues. Some methodological aspects could explain part of this problem, but another part could be attributable to a minor emphasis on these issues in the daily SUS management, which may be focused less on equity and more on increasing the production of and access to health care services. This problem has been partially compensated by the above-referenced academic research efforts, which placed high priority on the multidimensional issues related to equity in the Brazilian context [40,54,55,56,57,58].

Efforts to monitor NCDs and their relevant risk factors have become a point of emphasis in the past few years. These efforts include the best use of already existing data sources (death registrations, cancer registries, national surveys), initiatives such as VIGITEL [27], and new initiatives, such as the 1st National Health Survey (Pesquisa Nacional de Saude - PNS) [59], which uses a large population sample and includes measures and biological samples for examination.

Some efforts to integrate multiple indicators to create multidimensional indexes and/or integrated frameworks to guide monitoring and evaluative processes are in progress, although they are not conclusive; two of these efforts will be described here. One effort, the index of SUS performance (IDSUS), has been developed within the health system. This index is defined as a synthetic indicator and aims to be “a contextualized benchmarking of the performance of the Unified Health System (SUS) regarding access (potential or effective) and the effectiveness of primary care, outpatient, inpatient and emergency care” [60]. Other initiatives include the PROADESS (Performance Assessment of the Health System), which conceived a multilevel framework for evaluating the SUS, including the sub-dimensions of access, effectiveness, efficiency, appropriateness, continuity, safety, acceptability, and patient rights [61,62]; the Interagency Network for Health Information (RIPSA) [63]; and the Observatory on Inequities in Health [64].

The integrated approach and the results presented here and published elsewhere [1,51,65] confirm a view that SUS coverage is expanding overall, increasing access to all levels of care, with some important positive trends toward equity. This equity orientation has been clearly achieved in relation to relevant health determinants, in primary care and in some important health outcomes, and in a more limited way in secondary levels of health care. However, at the tertiary level of health care this reduction in inequality, if exists, it is not perceivable. It is possible that a group of factors could interfere with this dynamic [65]; tertiary care is dependent on more advanced technologies and resources that are spatially concentrated in developed and wealthier areas without the adequate configuration to reduce or eliminate geographic access as an obstacle to universal coverage and equity. The chronic underfunding of the SUS imposes serious limitations on the overall expansion of the SUS, particularly at the secondary and tertiary levels.

| **Box S4: Recommendations**  To strengthen monitoring and evaluation Unified Health System (SUS), defining a set of indicators interconnected by a consolidated framework that must reflect the several levels, components and effects of the unified heath system, relevant health determinants and health outcomes, with greater focus on equity;  Make of the efforts to the new generation of health information systems in development, including the individual SUS card, to be integrated in the monitoring and evaluation efforts;  Integrate to SUS health information systems relevant data on health care generated by private health plans;  To strengthen initiatives directed to expand and integrate primary health care (PSF) with secondary and tertiary care through the establishment of healthcare regions, underpinning the organisation of healthcare networks (hierarchy, territorialisation and coordination), making all the efforts to decrease social and geographical inequities;  Increase funding – guarantee through law enforcement, cut of public subsidies and investments in the private health sector. |
| --- |

**References**

1. Paim J, Travassos C, Almeida C, Bahia L, Macinko J. The Brazilian health system: history, advances, and challenges. *Lancet* 2011; published online May 9. DOI:10.1016/S0140-6736(11)60054-8.
2. Victora CG, Aquino EML, Leal MdC, et al. Maternal and child health in Brazil: progress and challenges. *Lancet* 2011; 377(9780):1863-76
3. Barreto ML, Teixeira MG, Bastos FI, et al. Successes and failures in the control of infectious diseases in Brazil: social and environmental context, policies, interventions, and research needs. *Lancet* 2011; 377(9780):1877-89.
4. Schmidt MI, Duncan BB, e Silva GA, et al. Chronic non-communicable diseases in Brazil: burden and current challenges. *Lancet* 2011; 377(9781):1949-61.
5. Reichenheim ME, de Souza ER, Moraes CL, et al. Violence and injuries in Brazil: the effect, progress made, and challenges ahead. *Lancet* 2011; 377(9781):1962-75
6. <http://data.worldbank.org/country/brazil> accessed in November 17^th^, 2013.
7. Instituto Brasileiro de Geografia e Estatística (IBGE). Pesquisa de Orçamentos Familiares 2008-2009. Despesas, Rendimentos e Condições de Vida. Rio de Janeiro: Instituto Brasileiro de Geografia e Estatística; 2010.
8. IPEA. Instituto de Pesquisa Econômica Aplicada (IPEA). A década inclusiva (2001–2011): desigualdade, pobreza e políticas de renda. Rio de Janeiro: IPEA, 2012.
9. Atlas do desenvolvimento Humano, PNUD-Brasil; Brasilia, 2013.
10. Teixeira MG, Costa MCN, Souza LPF, Nascimento EMR, Barreto ML, Barbosa N, Carmo EH. Evaluation of Brazil’s public health surveillance system within the context of the International Health Regulations (2005). *Rev Panam Salud Publica.* 2012;32(1):49–55.
11. Macinko J, Sousa MFM, Guanais F, Simões CS. Going to scale with community-based primary care: an analysis of the family health program and infant mortality in Brazil, 1999-2004. *Soc Sci Méd* 2007; **65:** 2070–80.
12. Aquino R, Oliveira NF, Barreto ML. Impact of the Family Health Program on infant mortality in Brazilian municipalities. *Am J Public Health* 2009; **1:** 87–93.
13. Rasella D, Aquino R, Barreto ML. Reducing childhood mortality from diarrhea and lower respiratory tract infections in Brazil. *Pediatrics* 2010; **126:** 534–40.
14. [Dourado I](http://lattes.cnpq.br/7845852622901449), Oliveira VB, Aquino R, Bonolo P, [Lima-Costa MF](http://lattes.cnpq.br/7404029442077952), [Medina MG](http://lattes.cnpq.br/9524524515892403), Mota E, Turci M, Macinko J. Trends in primary health care sensitive conditions in Brazil: the role of the Family Health Program (Project ICSAP-Brazil). Medical Care, 49: 577-584, 2011.
15. Guanais F, Macinko J. Primary care and avoidable hospitalizations: Evidence from Brazil. *J Ambul Care Manage* 2009; **32:** 114–21.
16. <http://dtr2004.saude.gov.br/susdeaz/pns/arquivo/Plano_Nacional_de_Saude.pdf>. . Accessed in 22/11/2013.
17. <http://www2.datasus.gov.br/DATASUS/index.php>. Accessed in 22/11/2013.
18. <http://portal.saude.gov.br/portal/saude/profissional/visualizar_texto.cfm?idtxt=32116>. Accessed in 22/11/2013.
19. <http://portal.saude.gov.br/portal/saude/visualizar_texto.cfm?idtxt=21379>. Accessed in 22/11/2013.
20. <http://dtr2004.saude.gov.br/sinanweb/>. Accessed in 22/11/2013.
21. <http://www2.datasus.gov.br/SIAB/index.php?area=01>. Accessed in 22/11/2013.
22. <http://sia.datasus.gov.br/principal/index.php>. Accessed in 22/11/2013.
23. <http://tabnet.datasus.gov.br/cgi/sih/midescr.htm>. Accessed in 22/11/2013.
24. <http://www.ibge.gov.br/home/estatistica/pesquisas/pesquisa_resultados.php?id_pesquisa=40>. Accessed in 22/11/2013.
25. <http://www.ibge.gov.br/home/estatistica/pesquisas/pesquisa_resultados.php?id_pesquisa=25> Accessed in 22/11/2013.
26. <http://portal.saude.gov.br/portal/saude/profissional/area.cfm?id_area=1521> Accessed in 22/11/2013.
27. WHO/WKC/Tech.Ser./04.2 A Glossary of Terms for Community Health Care and Services for Older Persons. WHO; Geneva, 2004
28. <http://www.pnud.org.br/atlas/ranking/Ranking-IDHM-Municipios-2000.aspx> Accessed in 22/11/2013.
29. Handbook on health inequality monitoring: with a special focus on low- and middle-income countries. World Health Organization, Geneva: 2013.
30. Chen ZA. CONCINDC: Stata module to calculate concentration index with both individual and grouped data. Available at: [http://econpapers.repec.org/software/ bocbocode/s456802.htm Accessed in 22/11/2013](http://econpapers.repec.org/software/%20bocbocode/s456802.htm%20Accessed%20in%2022/11/2013).
31. Lustig N, Lopez-Calva LF, Ortiz-Juarez. Declining Inequality in Latin America in the 2000s: the Cases of Argentina, Brazil, and Mexico. CGD Working Paper 307. Washington, DC: Center for Global Development. 2012. <http://www.cgdev.org/content/publications/detail/1426568> accessed in November 17^th^, 2013.
32. Brasil, Ministério da Integração Nacional. Programa Água Para Todos. http://www.integracao.gov.br/agua-para-todos Accessed in November 5^th^, 2013
33. Brasil, Ministério de Minas e Energia. Programa Luz para todos. http://luzparatodos.mme.gov.br/luzparatodos/asp/ Accessed in November 5^th^, 2013
34. Monteiro CA, Cavalcante TM, Moura EC, Claro RM, Szwarcwald CL. Population-based evidence of a strong decline in the prevalence of smokers in Brazil (1989–2003). Bull World Health Organ 2007; 85:527–34
35. Iglesias R, Jha P, Pinto M, Silva VL, Godinho J. Documento de discussão - saúde, nutrição e população (HNP) Controle do tabagismo no Brasil: resumo executivo. Epidemiol Serv Saude2008; 17:301–04
36. Macinko J, Dourado I, Aquino R, et al. Major Expansion Of Primary Care In Brazil Linked To Decline In Unnecessary Hospitalization. Health Aff 2010; 29: 2149–60.
37. Rasella D, Aquino R, Santos CA, Paes-Sousa R, Barreto ML. Effect of a conditional cash transfer programme on childhood mortality: a nationwide analysis of Brazilian municipalities. The Lancet 2013; 382: 57–64.
38. Guanais FC. The Combined Effects of the Expansion of Primary Health Care and Conditional Cash Transfers on Infant Mortality in Brazil, 1998-2010. Am J Public Health 2013; 103: 2000–6.
39. Macinko J, Lima-Costa MF. Horizontal equity in health care utilization in Brazil, 1998–2008. International Journal for Equity in Health 2012; 11: 33.
40. Barros FC, Matijasevich A, Requejo JH, et al. Recent trends in maternal, newborn, and child health in Brazil: progress toward Millennium Development Goals 4 and 5. Am J Public Health 2010; 100: 1877–89.
41. Barata RB, Ribeiro MCS de A, de Moraes JC, Flannery B, Vaccine Coverage Survey 2007 Group. Socioeconomic inequalities and vaccination coverage: results of an immunization coverage survey in 27 Brazilian capitals, 2007-2008. J Epidemiol Community Health 2012; 66: 934–41.
42. Martínez-Mesa J, Werutsky G, Campani RB, Wehrmeister FC, Barrios CH. Inequalities in Pap smear screening for cervical cancer in Brazil. Prev Med 2013; 57: 366–71.
43. Lee BL, Liedke PE, Barrios CH, Simon SD, Finkelstein DM, Goss PE. Breast cancer in Brazil: present status and future goals. Lancet Oncol 2012; 13: e95–e102.
44. Barros AJD, Santos IS, Matijasevich A, et al. Patterns of deliveries in a Brazilian birth cohort: almost universal cesarean sections for the better-off. Rev Saude Publica 2011; 45: 635–43.
45. Bassanesi SL, Azambuja MI, Achutti A. Premature mortality due to cardiovascular diseases and social inequalities in Porto Alegre: from evidence to action. Arq Bras Cardiol2008;90:370–9
46. Zerbini E de J. A cirurgia cardiovascular no Brasil: realizações e possibilidade. Revista Brasileira de Cirurgia Cardiovascular 2010; 25: 264–77.
47. Machado EL, Caiaffa WT, César CC, et al. Iniquities in the access to renal transplant for patients with end-stage chronic renal disease in Brazil. Cadernos de Saúde Pública 2011; 27: s284–s297.
48. Garcia LP, Sant’Anna AC, Magalhães LCG, Aurea AP. Gastos com saúde das famílias brasileiras residentes em regiões metropolitanas: composição e evolução no período 1995-2009. Ciência e Saúde Coletiva, 18(1): 115-128, 2013.
49. Diniz BPC, Servo LMS, Piola SF, Eirado M. Gasto das famílias com saúde no Brasil: evolução e debate sobre gasto catastrófico. Gasto e consumo das famílias brasileiras contemporâneas [Internet]. Brasília: IPEA; 2007 [acessado 2013, set]. Disponível em: http://www.ipea.gov.br/sites/000/2/livros/gastoeconsumov2/10_Cap04.pdf
50. Victora CG, Barreto ML, do Carmo Leal M, Monteiro CA, Schmidt MI, Paim J, Bastos FI, Almeida C, Bahia L, Travassos C, Reichenheim M, Barros FC; Lancet Brazil Series Working Group. Health conditions and health-policy innovations in Brazil: the way forward. Lancet. 2011 Jun 11;377(9782):2042-53.
51. Santos LM, Souza LE, Serruya SJ, Guimarães RF. The role of research in the consolidation of the Unified National Health System (SUS). Cad Saude Publica. 2010 Sep;26(9):1666-7.
52. <http://portal.saude.gov.br/portal/saude/Gestor/area.cfm?id_area=944> Accessed in 22/11/2013.
53. Barata RB, de Almeida MF, Montero CV, da Silva ZP. Health inequalities based on ethnicity in individuals aged 15 to 64, Brazil, 1998. Cad Saude Publica. 2007 Feb;23(2):305-13.
54. Pinheiro, R. S., Viacava, F., Travassos, C., & Brito, A. D. S. (2002). Gênero, morbidade, acesso e utilização de serviços de saúde no Brasil. *Ciência & Saúde Coletiva*, *7*(4), 687-707.
55. Viana, S. M., Nunes, A., Santos, J. R. S., & Barata, R. B. (2001). Medindo as desigualdades em saúde no Brasil: uma proposta de monitoramento. *Brasília: Organização Pan-Americana da Saúde/Instituto de Pesquisa Econômica Aplicada*.
56. Travassos, C., Viacava, F., Fernandes, C., & Almeida, C. M. (2000). Desigualdades geográficas e sociais na utilização de serviços de saúde no Brasil. *Cienc saúde coletiva*, *5*(1), 133-49.
57. Rasella D, Aquino R, Barreto ML. Impact of income inequality on life expectancy in a highly unequal developing country: the case of Brazil. J Epidemiol Community Health. 2013 Aug;67(8):661-6.
58. <http://www.pns.icict.fiocruz.br/> Accessed in 22/11/2013.
59. [http://portal.saude.gov.br/portal/saude/area.cfm?id_area=1080](http://portal.saude.gov.br/portal/%20saude/area.cfm?id_area=1080). Accessed in 22/11/2013.
60. <http://www.proadess.icict.fiocruz.br/>. Accessed in 22/11/2013.
61. Viacava F, Ugá MA, Porto S, Laguardia J, Moreira Rda S. [Evaluation of performance of health systems: a model for analysis]. Cien Saude Colet. 2012 Apr;17(4):921-34)
62. <http://www.ripsa.org.br/php/index.php> Accessed in 22/11/2013.
63. <http://dssbr.org/site/> Accessed in 22/11/2013.
64. Fundação Oswaldo Cruz. A saúde no Brasil em 2030: diretrizes para a prospecção estratégica do sistema de saúde brasileiro. / Fundação Oswaldo Cruz... [et al.]. Rio de Janeiro : Fiocruz/Ipea/Ministério da Saúde/Secretaria de Assuntos Estratégicos da Presidência da República, 2012.
